# Excessive Venery, Masturbation and Continence

**Published:** 1888-10

**Authors:** 


**Excessive Venery, Masturbation and Continence.** The Etiology, Pathology and Treatment of the Diseases resulting from Venereal Excesses, Masturbation and Continence. By Joseph W. Howe, M. D., late Professor of Clinical Surgery in the Bellevue Hospital Medical College; author of "Emergencies," etc., etc. Second edition, revised; 8vo, pp. 300. New York: E. B. Treat, publisher. Cloth. Price, $2.75.

This is an important work on a difficult subject. Perhaps there are no other questions coming before the physician for decision more perplexing than those concerning treatment of the class of diseases of which this book treats. It is to be appreciated, therefore, that Dr. Howe has written a work which is thoroughly reliable, its matter judiciously presented, and which shows that it contains the experience of a practical surgeon of broad views and large opportunities of observation. His advice is often bold, but never rash or ill-considered. It is recognized that these questions must be treated from a religious and moral standpoint as well as from a physiological and pathological one. As far as we know, it is the only work that is satisfactory from these various points of view. It discusses the hygiene of the sexual organs; their physiology; the diseases arising from excesses, from continence, from masturbation; it considers the influence of the mind and social surroundings; and, finally, the chapters on treatment are elaborate and full. These chapters contain not only the author's views, but also a summary of the views of others who have written on this subject. The work is, therefore, useful as a book of reference, which increases its value. While it is well made up, there are a few typographical errors that should be corrected. At the top of page nineteen there is a whole line that seems to have no relation to the text. We feel sure there will be plenty of opportunities to correct these, as the book should be, and doubtless will be, widely read, and this will necessitate further editions. We commend it to all who desire to be thoroughly familiar with this class of cases, especially with their causes and treatment.

